# Abrogation of PRRSV infectivity by CRISPR-Cas13b-mediated viral RNA cleavage in mammalian cells

**DOI:** 10.1038/s41598-020-66775-3

**Published:** 2020-06-15

**Authors:** Jin Cui, Navapon Techakriengkrai, Teerawut Nedumpun, Sanipa Suradhat

**Affiliations:** Department of Veterinary Microbiology, Faculty of Veterinary Science, Chulalongkorn University, Pathumwan, Bangkok, 10330 Thailand

**Keywords:** RNA, Genetic vectors, Antivirals

## Abstract

CRISPR/Cas9 enables dsDNA viral genome engineering. However, the lack of RNA targeting activities limits the ability of CRISPR/Cas9 to combat RNA viruses. The recently identified class II type VI CRISPR/Cas effectors (Cas13) are RNA-targeting CRISPR enzymes that enable RNA cleavage in mammalian and plant cells. We sought to knockdown the viral RNA of porcine reproductive and respiratory syndrome virus (PRRSV) directly by exploiting the CRISPR/Cas13b system. Effective mRNA cleavage by CRISPR/Cas13b-mediated CRISPR RNA (crRNA) targeting the ORF5 and ORF7 genes of PRRSV was observed. To address the need for uniform delivery of the Cas13b protein and crRNAs, an all-in-one system expressing Cas13b and duplexed crRNA cassettes was developed. Delivery of a single vector carrying double crRNAs enabled the simultaneous knockdown of two PRRSV genes. Transgenic MARC-145 cells stably expressing the Cas13b effector and crRNA mediated by lentiviral-based transduction showed a robust ability to splice the PRRSV genomic RNA and subgenomic RNAs; viral infection was almost completely abrogated by the combination of double crRNAs simultaneously targeting the ORF5 and ORF7 genes. Our study indicated that the CRISPR/Cas13b system can effectively knockdown the PRRSV genome *in vitro* and can potentially be used as a potent therapeutic antiviral strategy.

## Introduction

RNA viruses remain a great threat to humans and animals around the world^1,2^. Vaccination is the primary strategy to prevent viral infections in hosts^3^. Unlike DNA viruses, the extremely high evolutionary rates of RNA viruses have led to the rapid emergence of variant virus strains^4,5^. Heterologous infections often cause vaccine failure. In addition, vaccines against many RNA viruses have not been successfully developed^6^. Following viral infection, antiviral drug treatments are the only clinical therapy to combat viruses. However, there are only a limited number of antivirals available targeting viruses that threaten public human health, such as HIV, HBV, HCV, herpesviruses and influenza viruses^7^. Although certain compounds have the potential to be developed as broad-spectrum antiviral agents, the relatively high cost limits their widespread clinical use in veterinary medicine, RNA viral infection causes significant economic loss in the livestock industry.

Porcine reproductive and respiratory syndrome (PRRS) has been one of the most important infectious viral diseases in swine worldwide for almost three decades^8,9^. Porcine reproductive and respiratory syndrome virus (PRRSV), the aetiological agent of PRRS, is an enveloped single-stranded positive-sense RNA virus belonging to the family *Arteriviridae*^10^. Presently, PRRSV is taxonomically classified into the genus *Porarterivirus*^11,12^. The PRRSV genome is approximately 15 kb in length with at least 10 open reading frames (ORFs)^13^. PRRSV utilizes two distinct transcription mechanisms to express viral accessory and structural proteins^14^. The two-third 5′ terminal PRRSV genome contains two replicase-associated genes (ORF1a and ORF1b) encoding polyproteins that are further processed into 17 non-structural proteins (NSPs) responsible for PRRSV transcription and replication^15,16^. The PRRSV structural proteins are produced by a nested set of 3′ co-terminal subgenomic RNAs^17^. PRRSV consists of two highly heterologous species, namely, PRRSV-1 (formerly European genotype 1) and PRRSV-2 (formerly North American genotype 2)^12,18^. The PRRSV-1 species is classified into three subtypes (subtypes 1–3) and the PRRSV-2 species is further divided into typical and atypical strains^19–21^. The PRRSV-1 Lena strain (subtype 3) and PRRSV-2 atypical strains have been described as highly pathogenic PRRSV strains characterized by high fever and high mortality rates in pigs of all ages^19,22^. PRRSV can suppress the host immune system, which may allow secondary/opportunistic pathogens to establish infections, resulting in more severe and chronic diseases^23^. Due to extensive antigenic variability and immunomodulatory properties of the virus, control of PRRSV has proven to be a challenge throughout the world. Therefore, the development of alternative and effective treatment strategies is urgently needed.

The clustered regularly interspaced short palindromic repeats (CRISPR) and CRISPR-associated (Cas) systems provide adaptive immunity to bacteria and archaea, enabling organisms to respond to and eliminate invading foreign genetic elements^24,25^. The CRISPR-Cas systems form two major classes, namely, class 1 and class 2 which employ multiple Cas proteins and a single large Cas protein for nucleic acid interference respectively^24,26^. Considering the simple effector structure, class 2 CRISPR-Cas systems have been widely engineered as genome-editing tools^27,28^. Class 2 consists of three types (type II, V and VI) and can be further divided into several subtypes^29–31^. Among them, type II Cas9 is the most commonly studied CRISPR effector for double-stranded DNA (dsDNA) engineering^27,32,33^. CRISPR RNA (crRNA) leads the Cas9 protein to recognize and bind target sites and then results in a double-strand break (DSB) approximately 3 bp upstream of the protospacer adjacent motif (PAM) through the RuvC and HNH nuclease domains^34,35^. The DSBs can be subsequently repaired by the homology-directed repair (HDR) pathway which requires a donor template or the non-homologous end joining (NHEJ) pathway in the absence of a homologous template within the target DNA for genome editing purposes^36,37^.

Recently, type VI Cas effectors (Cas13) were identified as RNA-targeting CRISPR enzymes enabling RNA knockdown in mammalian and plant cells displaying high specificity and targeting flexibility^38–40^. In contrast to Cas9, the HNH and RuvC domains do not exist in Cas13 proteins. The dual higher eukaryotes and prokaryotes nucleotide (HEPN)-binding endoRNase domains of Cas13 mediate the precise cleavage of targeted transcripts^41–44^. The splicing activity varies in different Cas13 subtypes and orthologues. By comparison of 21 Cas13a, 15 Cas13b and 7 Cas13c orthologues, the Cas13b orthologue from *Prevotella* sp. P5–125 (PspCas13b) proved to be the most robust and specific for RNA knockdown in mammalian cells^40^. In addition, the most recently discovered Cas13d was more efficient than PspCas13b^38^. Importantly, type VI enzyme-mediated RNA cleavage shows no PAM preference in eukaryotes, allowing flexible design of potential targeting sites^38–40^. In bacteria, Cas13 exhibits nonspecific cleavage of any transcripts near the target and activates programmed cell death to limit bacteriophage infections^39,43^. Fortunately, such collateral RNA degradation activity is not present in mammalian and plant cells, allowing specific RNA targeting^38–40^. Furthermore, Cas13 exhibits a high specificity of RNA interference activity relative to RNA interference (RNAi), as no off-target effects were detectable in eukaryotic cells^38,40^. In addition to RNA knockdown capability, fusion of catalytically inactive Cas13 (dCas13) to an RNA-editing enzyme retained the RNA-binding activity and enabled specific RNA editing in mammalian cells^40^. These advantages of type VI CRISPR systems have the potential to be developed as a platform to combat RNA viruses by targeting and degrading viral RNA in mammalian cells.

Here, we sought to investigate the possibility of adopting the CRISPR/Cas13b system for interference against PRRSV RNA in eukaryotic cells. To this end, we designed multiple specific crRNAs targeting the PRRSV essential genes ORF5 and ORF7. Our study revealed that the CRISPR/Cas13b catalytic activities resulted in interference with PRRSV gene transcription and expression. Furthermore, a novel all-in-one CRISPR/Cas13b delivery system that enabled cells to be transfected with the Cas13b effector and duplex-targeting guides simultaneously was established. The CRISPR/Cas13b system enabled an almost complete knockdown of PRRSV genomic and subgenomic RNAs to eradicate viral infections in lentiviral-mediated transgenic MARC-145 cells expressing Cas13b and duplexed crRNAs. Our study indicated the potential of using the CRISPR/Cas13b system as a novel therapeutic strategy by directly targeting the viral genes of RNA viruses.

## Results

### Characterization of the CRISPR/Cas13b system in PRRSV mRNA targeting

**Figure 1:**
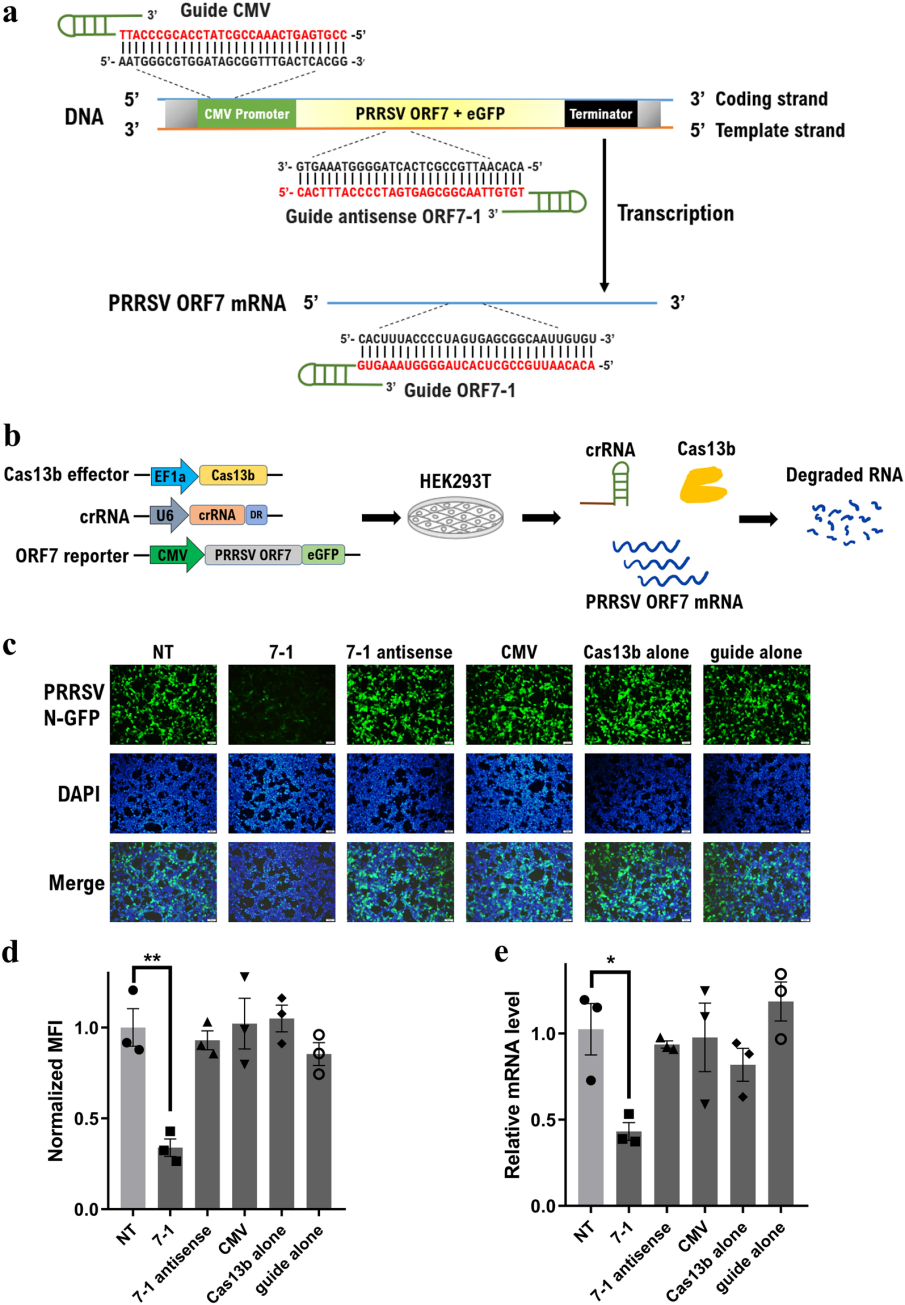
Characterization of the CRISPR/Cas13b system in PRRSV mRNA targeting. (**a**) Schematic diagram of the design of crRNAs targeting the ORF7 mRNA transcript, template strand of the ORF7 gene and CMV promoter. (**b**) Schematic diagram showing the steps of the determination of the effect of CRISPR/Cas13b on PRRSV gene knockdown by co-transfection of the three plasmids into HEK293T cells. (**c**) Microscopic fluorescence images showing the expression of the PRRSV ORF7-eGFP reporter after CRISPR/Cas13b activity with various targeting crRNAs. The bar indicates 100 μm. (**d**,**e**) PRRSV N protein expression and ORF7 mRNA levels were determined by flow cytometry and quantitative RT-PCR, respectively. Values shown as the mean ± SEM with n = 3. *and **refer to P values < 0.05 and 0.01, respectively.

We aimed to explore whether the CRISPR/Cas13b system could directly knocking down PRRSV mRNA. Initially, the PRRSV ORF7 gene encoding the nucleocapsid (N) protein, which is the main component of the viral capsid encapsulating viral RNA and is involved in the regulation of host cell processes^45^, was selected as the target. A PRRSV ORF7 gene fused with an eGFP reporter plasmid was generated, and a set of guide RNA targeting sequences was designed and then inserted into the CRISPR/Cas13b guide RNA backbones (Fig. 1a). To assess whether the ORF7 mRNA could be repressed by CRISPR/Cas13b targeting, HEK293T cells were simultaneously co-transfected with Cas13b, ORF7-eGFP and individual crRNA (Fig. 1b). At 48 h post-transfection, the expression of the PRRSV N protein was analysed. The expression levels of viral protein upon specific ORF7 crRNA targeting were dramatically lower than those of the nonspecific guide RNA control group (Fig. 1c). Flow cytometric analysis revealed that the mean fluorescence intensity decreased by 66.1% (Fig. 1d). To further monitor Cas13b-mediated cleavage of the ORF7 transcript, quantitative RT-PCR was performed, and CRISPR/Cas13b knocked down approximately 56.9% of the mRNA transcripts (Fig. 1e). Conversely, delivery of either the specific ORF7 guide RNA or Cas13b alone had no effect on ORF7 mRNA knockdown (Fig. 1c–e), demonstrating efficient CRISPR/Cas13b RNA targeting. Additionally, no inhibitory effects were detected when cells were treated with guides targeting the CMV promoter or template strand of the ORF7 gene (Fig. 1c–e), indicating that CRISPR/Cas13b specifically knocked down mRNA rather than dsDNA.

**Figure 2:**
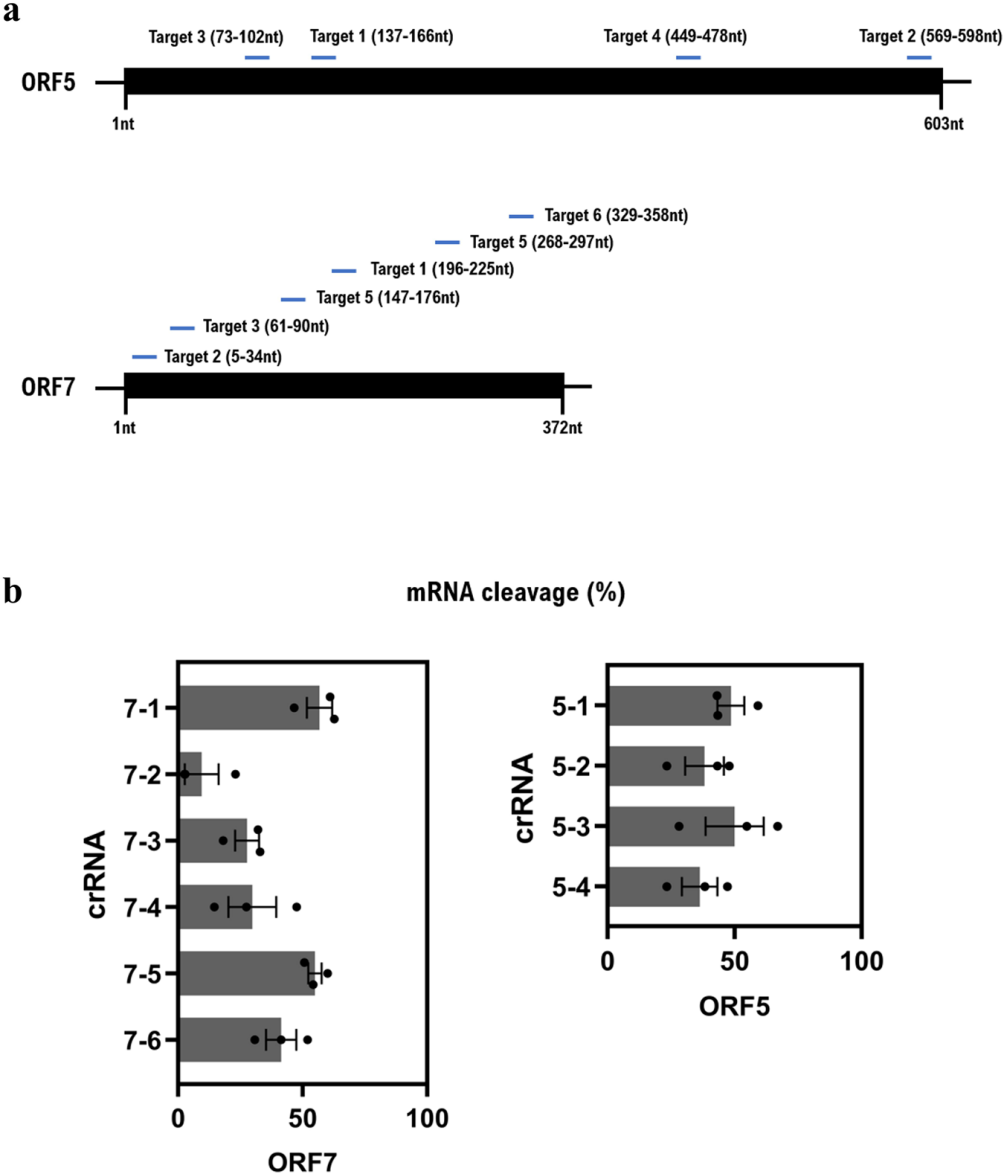
Determination of the most potent crRNA for Cas13b-mediated PRRSV ORF5 and ORF7 targeting. (**a**) Locations of crRNA targeting regions within the ORF5 and ORF7 genes. (**b**) RNA cleavage efficiency was determined for the indicated crRNAs targeting ORF5 and ORF7 by qRT-PCR and normalized to the non-targeting (NT) control.

We subsequently determined the most potent guide RNAs for Cas13b-mediated PRRSV targeting. We also employed another PRRSV gene, ORF5, which is the major viral envelope protein and inducer of neutralizing antibodies *in vivo*^46^, for screening. Six and four regions were selected to target the ORF7 and ORF5 genes, respectively (Fig. 2a, Table 1). Varying degrees of gene repression were observed (Fig. S1). The most potent guides to ORF7 and ORF5 both led to approximately 50% gene knockdown efficiency (Fig. 2b. 50.02% and 56.85% for crRNA 5-1 and 7-1, respectively).

**Table 1:** The crRNA sequences used in this study.

crRNA	Target gene/region	Spacer sequence (5′ to 3′)
5-1	PRRSV ORF5	GACGACCCCATTGTTCCGCTGAAACTCTGG
5-2	PRRSV ORF5	GCCAATCTGTGCCATTCAGCTCACATAGCG
5-3	PRRSV ORF5	GCTGTTGCTGGCGTTGGCGAGCACAGCAAG
5-4	PRRSV ORF5	TGACGGGTGACCGCCAACGATAGAGTCTGC
7-1	PRRSV ORF7	ACACAATTGCCGCTCACTAGGGGTAAAGTG
7-2	PRRSV ORF7	TTCTCTTTTGCTGCTTGCCGTTGTTATTTG
7-3	PRRSV ORF7	GATGATCTTACCCAGCATTTGGCACAGCTG
7-4	PRRSV ORF7	GTCGCTAGAGGGAAATGGGGCTTCTCCGGG
7-5	PRRSV ORF7	ACTTATTCTCCCTGAATCTGACAGGGTACA
7-6	PRRSV ORF7	ATGCTGTGGCGCGGATCAGACGCACAGTAT
CMV	CMV promoter	CCGTGAGTCAAACCGCTATCCACGCCCATT
7-1 antisense	Antisense of PRRSV ORF7 mRNA	CACTTTACCCCTAGTGAGCGGCAATTGTGT
NT	Non-targeting	ATGTCTTCCTGGGACGAAGACAA

### Development of a single CRISPR/Cas13b-duplexed guide RNA delivery system

**Figure 3:**
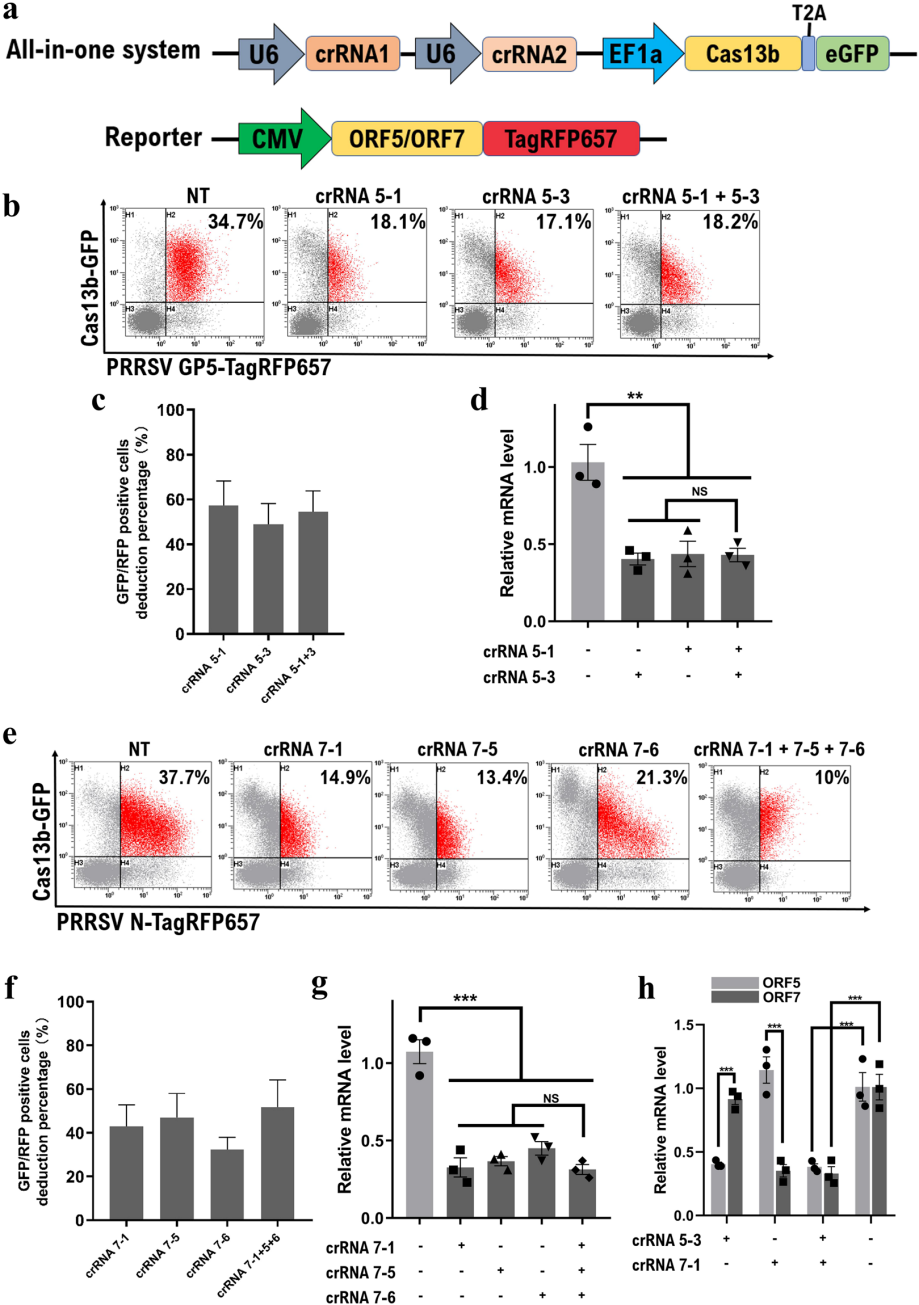
Development of an all-in-one CRISPR/Cas13b-duplexed crRNA delivery system. (**a**) The structure of the all-in-one delivery vector and PRRSV gene reporter. The U6 promoter drives duplexed guide expression, and the EF1a promoter mediates the transcription of Cas13b. The eGFP reporter fused with Cas13b by a 2 A self-cleaving peptide facilitates the detection of Cas13b expression. (**b**,**c**,**d**) The all-in-one Cas13b system carrying specific PRRSV ORF5 crRNA significantly cleaved ORF5 mRNA. However, no accumulative effect of Cas13b was detected by targeting the ORF5 gene with two crRNAs simultaneously (**b**,**c**), flow cytometry; (**d)**, real-time PCR). (**e**) The all-in-one plasmid was further modified to incorporate triple crRNAs targeting the ORF7 gene. Representative flow cytometry graphs for eGFP- and RFP657-positive cells are shown. (**f**) The reduction percentages of dual-marker positive cells were determined by flow cytometry. Each crRNA group was normalized to the NT control. (**g**) The percentage of RNA reduction was determined by qRT-PCR. (**h**) The established platform enables the simultaneous knockdown of PRRSV ORF5 and ORF7 mRNA by incorporating two corresponding guide protospacer sequences. Values are shown as mean ± SEM with n = 3. **, ***and NS refer to P values < 0.01, 0.001 and no significant differences, respectively.

To improve the efficacy of the CRISPR/Cas13b system, a single CRISPR/Cas13b-duplexed crRNA delivery system was developed in this study. The vector consisted of a Cas13b effector and two guide crRNAs. Cas13b was co-expressed with an eGFP reporter gene to facilitate the determination of transfection efficiency. Two guide RNA cassettes with Cas13b direct repeats driven by individual U6 promoters were inserted upstream of Cas13b to facilitate co-expression with the Cas13b protein (Fig. 3a). A Golden Gate assembly method was developed to facilitate the rapid and efficient cloning of single- or double-crRNA sequences into the all-in-one system (Fig. S2).

Initially, we showed that eukaryotic cells effectively expressed Cas13b along with dual crRNAs by the established all-in-one platform delivery system (Fig. S3). To further characterize the constructed system, we tested the possibility of streamlined duplexed delivery of guide RNAs targeting a single PRRSV gene. The crRNA 5-1 and 5-3 constructs targeting ORF5 were inserted into the all-in-one vector by Golden Gate assembly. Additionally, either crRNA 5-1 or 5-3 was replaced by non-targeting crRNA as a single, specific crRNA control. Upon transfection of HEK293T cells with the all-in-one platform and an ORF5-RFP657 reporter, a loss of approximately 50% of the eGFP and RFP657 co-expression cells carrying either the crRNA 5-1 or 5-3 single guide was detected (Fig. 3b,c). However, the introduction of a combination of two crRNAs did not induce a further reduction in RFP657 expression (Fig. 3b,c). We assessed the ORF5 mRNA content in these crRNA-expressing cells, and a slightly higher knockdown degree (approximately 60%, Fig. 3d) was observed in cells treated with the all-in-one system than in the cells treated with the individual CRISPR/Cas13b component delivery system (approximately 50%, Fig. 2b). However, consistent with the flow cytometry results, Cas13b lacked accumulative activity on the ORF5 gene by duplexed crRNA targeting. To further address this issue, we modified the overhangs within the all-in-one plasmids to enable triple crRNAs targeting. When the three crRNAs targeting ORF7 were delivered into cells, a mildly greater reduction in RFP657 expression was observed in Cas13b-expressing populations than in each single crRNA-expressing group (Fig. 3e,f). However, no significant differences in the ORF7 mRNA were observed between cells carrying single crRNAs and triple crRNAs (Fig. 3g). These results indicated that the multiplexed targeting approach did not improve RNA cleavage in the current all-in-one system.

Therefore, we questioned whether the all-in-one system enabled the simultaneous knockdown of two PRRSV genes. To this end, we inserted the most potent crRNAs for ORF5 and ORF7 targeting into the cassettes; the all-in-one Cas13b-crRNA 5-3 + 7-1 along with a combination of separate ORF5 and ORF7 reporters was delivered into HEK293T cells. As shown in Fig. 3h, compared to the NT crRNA group, the double-crRNA-expressing cell group exhibited a significantly decrease in both the ORF5 and ORF7 mRNA levels (Fig. 3h). To evaluate the specificity of the system, each of the two guides was replaced with an NT guide. In each case where a specific guide was absent from the cassettes, only the targeted transcripts were reduced (Fig. 3h), indicating that the all-in-one system is capable of simultaneously knocking down two genes by a combination of two corresponding crRNAs.

### Lentiviral-mediated CRISPR/Cas13b delivery eradicates PRRSV infection *in vitro*

**Figure 4:**
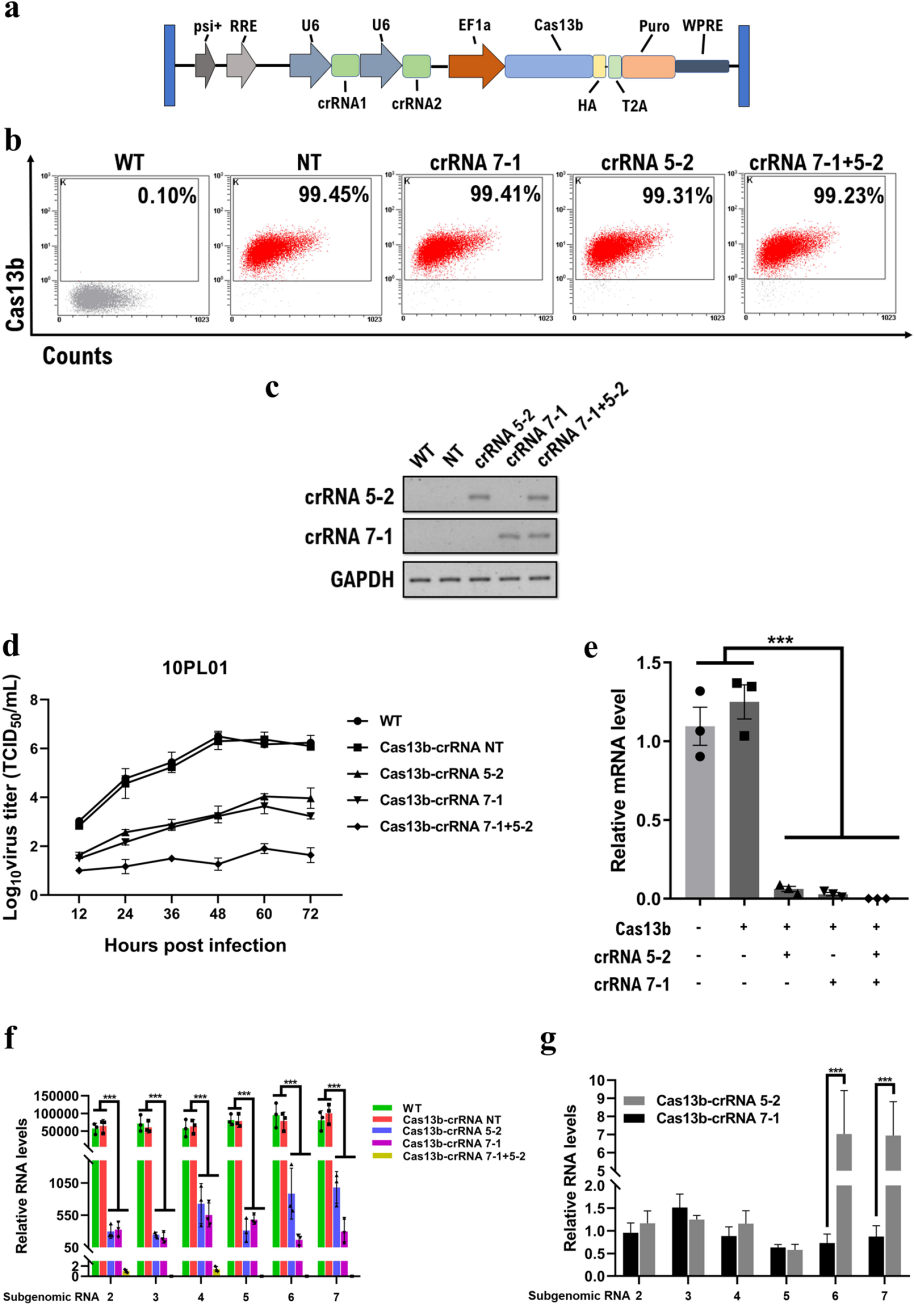
Cas13b mediates the efficient knockdown of the PRRSV genome in lentiviral transgenic MARC-145 cells. (**a**) Schematic diagram for lentiviral transfer gene constructs encoding Cas13b and crRNAs. (**b**) Determination of Cas13 effector expression levels in puro-selected transgenic cells by flow cytometry analysis. (**c**) The expression of corresponding crRNAs in each cell line was detected by PCR. PCR products were separated by 5% agarose gels. (**d**) The growth kinetics of HP-PRRSV strain 10PL01 with an MOI of 0.1 in transgenic cells. (**e**) The Cas13b cleavage activity on PRRSV genomic RNA was determined by qRT-PCR with primers targeting the NSP9 gene. (**f**) The PRRSV subgenomic RNA levels were measured by qRT-PCR with a set of specific primers targeting each subgenomic RNA. (**g**) Comparison of each subgenomic RNA knockdown efficiency between cells expressing Cas13b-crRNA 5-2 and Cas13b-crRNA 7-1. Values are shown as the mean ± SEM with n = 3. ***refers to P value < 0.001.

We next assessed the anti-PRRSV activity of the CRISPR/Cas13b-gRNA system in transgenic cells. Due to the low transfection efficiency in MARC-145 cells, we generated four lentiviral transfer plasmids carrying Cas13b along with various crRNAs (Fig. 4a). The 5-2 and 7-1 crRNAs were selected for targeting ORF5 and ORF7, respectively. Flow cytometric analysis confirmed the high expression of the Cas13b effector in all puromycin-selected cell lines (Fig. 4b). PCR results verified that each cell line expressed the corresponding crRNA (Fig. 4c).

Since the targeting regions between HP-PRRSV (strain 10PL01) and the classic PRRSV (strain 01NP1) were identical (data not shown), we determined the effect of the CRISPR/Cas13b system on the viral infectivity of both strains. Initially, the cells were infected with the HP-PRRSV strain 10PL01 and the classic strain 01NP1 at an MOI of 0.1. The culture medium was harvested every 12 h to determine the viral growth kinetics. The MARC-145 cells containing Cas13b and specific PRRSV crRNAs displayed high levels of virus resistance (Fig. 4d, Fig. S4). Either the single 5-2 guide or the 7-1 guide cell line showed a significantly decreased susceptibility to both PRRSV strains with an approximate 3-log reduction in virus titers at 48 hours post-infection. Surprisingly, the duplexed crRNA-expressing cell line showed almost complete inhibition of PRRSV infections. In contrast, cells stably expressing Cas13b and NT gRNA showed similar susceptibility to PRRSV during the whole viral infection cycle compared with parental MARC-145 cells (Fig. 4d, Fig. S4). Additionally, growth kinetic studies at a PRRSV MOI of 0.1 or 1 showed comparable results (data not shown).

To investigate CRISPR/Cas13b knockdown activities on PRRSV genomic RNA of the 10PL01 strain, we performed qRT-PCR to detect NSP9 gene, which does not exist in any subgenomic RNAs encoding viral structural proteins. Consistent with the viral growth kinetic studies, compared with the two control cell lines, MARC-145 cells expressing crRNA 5-2 and 7-1 showed strong PRRSV genomic RNA splicing with reductions of 93.8% and 97.0%, respectively (Fig. 4e). Combining the two crRNAs targeting ORF5 and ORF7 resulted in an almost complete loss of genomic RNA (over 99.99% reduction). A parallel tendency was also observed in PRRSV viral subgenomic RNAs (Fig. 4f). Cells expressing Cas13b along with dual crRNAs showed robust PRRSV subgenomic RNA knockdown activity, and most subgenomic RNAs remained at undetectable levels. The ORF7 transcript exists in all six subgenomic RNAs, but only the subgenomic RNA 2-5 contains the ORF5 transcript. Interestingly, cells expressing Cas13b-gRNA 5-2 showed a high reduction in all subgenomic RNAs (Fig. 4f), including subgenomic RNA 6 and 7, most likely due to the efficient knockdown of genomic RNA. As the two single-crRNA cell lines exhibited similar resistance to PRRSV, we specifically compared each subgenomic RNA level in two cell lines at 12 hours post-infection. As expected, subgenomic RNA 2–5 showed comparable levels in the two cell lines; however, subgenomic RNA 6 and 7 levels were significantly decreased in Cas13b-crRNA 7–2 cells compared with those in Cas13b-crRNA 5-2 cells (Fig. 4g), indicating the high target-specificity of CRISPR/Cas13b knockdown activities in transgenic cells. Taken together, these results indicate that lentiviral-based transduction mediated robust CRISPR/Cas13b RNA cleavage of both PRRSV genomic RNA and subgenomic RNAs in mammalian cells. The combination of dual crRNAs targeting two different viral genes was sufficient to abrogate PRRSV infection *in vitro*.

## Discussion

CRISPR/Cas technologies are powerful tools for gene therapy^47–49^. Multiple dsDNA viruses have been subjected to antiviral CRISPR/Cas9 targeting *in vitro* and *in vivo*, including EBV, HSV-1, HCMV^50^, HBV^51^ and HPV^52^. During a viral infection, there are two main strategies to inhibit the virus by using CRISPR/Cas9 technology: (i) targeting host genes that are essential for virus infection and (ii) targeting viral DNA to directly eliminate viral replication^53^. The major obstacle for the application of the CRISPR system in RNA virus infections is that most functionally characterized systems target dsDNA rather than RNA. Therefore, previous studies selected the host essential genes CCR5 and CXCR4, which are the chemokine co-receptors responsible for viral entry for HIV infection, as targets^54–56^. Alternatively, by selectively disrupting HIV proviral DNA integrated into the host genome, some groups have demonstrated that targeting HIV long terminal repeats (LTRs) or essential viral genes is an effective approach to profoundly suppress HIV production *in vitro*^57–62^ and *in vivo*^63^. However, there are no dsDNA intermediates in the majority of RNA viruses, and host essential genes are the only choice for CRISPR/Cas9 targeting, including for TGEV^64^, IAV^65^, CSFV^66^, FMDV^67^ and BVDV^68^. A recent study described the application of CRISPR/Cas9 to pig zygotes, resulting in the generation of pigs with a deletion of exon 7, which encodes SRCR5 of the CD163 gene responsible for binding to GP4 to mediate viral entry. These pigs showed complete resistance to type 1 PRRSV infections^69^. Although the size, stature and other morphological features of these pigs were comparable to those of wild-type pigs, the long-term impact of the disruption of functional cellular proteins remains uncertain.

The Cas13 enzyme is the first CRISPR-based system to enable precise RNA targeting and editing. The antiviral activities of type VI genotype have been determined in plants^70^. In plants, the Cas13a orthologue from *Leptotrichia shahii* (LshCas13a) showed approximately a 50% RNA interference with turnip mosaic virus (TuMV)^70^. The mild inhibitory efficacy is probably due to the less robust RNA knockdown ability of Cas13a than that of Cas13b. During the revision process of this manuscript, the same group further demonstrated that LwaCas13a, PspCas13b and CasRx variants (Cas13d) could mediate high interference activities against plant RNA viruses. Moreover, Cas13d exhibited the most robust interference ability in plants^71^. Meanwhile, several studies had focused on implementing CRISPR/Cas13-based strategies on RNA viruses, including the SARS-CoV-2 virus^72,73^.

In this study, we demonstrated that CRISPR/Cas13b with specific guide RNAs enabled the targeting and cleavage of the PRRSV ORF5 and ORF7 genes in eukaryotic cells. Conversely, the guides targeting either the CMV promoter, which is a dsDNA, or the antisense mRNA of the PRRSV genes failed to knockdown the mRNA transcripts, suggesting that the Cas13b effector specifically selected single-stranded RNA as a substrate. In bacteria, a double-sided protospacer flanking sequence (PFS) has been shown to affect Cas13b activity^44^, whereas PFS constraints are absent in mammalian cells^40^. CRISPR/Cas13 tolerates one mismatch between target RNA and crRNA, and the existence of more than two mismatches decreases the cleavage activity^43^. The optimal Cas13b targeting condition is not fully understood. A previous study indicated that the Cas13 binding and cutting efficiency were related to the secondary structure of the target RNA^43,74,75^. The low homology between PRRSV-1 and PRRSV-2 makes it difficult to design crRNAs targeting both PRRSV species within the ORF5 or ORF7 gene (Fig. S5a,b). Although most of the crRNAs shared high similarity with the majority of PRRSV-2 strains (Fig. S5c to l), this does not guarantee that the tested crRNAs were applicable to all PRRSV-2 strains. The heterology of non-targeting regions between PRRSV-2 strains may have an impact on viral RNA structures, altering the Cas13 cutting efficiency. A web service for Cas13a and Cas13d guide design is recently available^76,77^. However, considering the fast evolutionary rates of RNA viruses and the lack of a Cas13b-specific guide design tool, selection of multiple crRNAs against each targeted gene is necessary.

One of the major challenges for implementing the CRISPR/Cas-based genome engineering system is the efficient and simultaneous delivery of the Cas protein and guide RNAs. The Cas protein and guide RNA have been introduced into cells mostly via independent plasmids by co-transfection. Co-transfection of multiple plasmids leads to variable expression of CRISPR components in each cell. To overcome this problem, we developed a single CRISPR/Cas13b-duplexed crRNA delivery system that efficiently expressed the Cas13b effector and dual or multiple mature guide RNAs. In addition, this system included an eGFP marker to indicate Cas13b expression. Although no accumulative effect of Cas13b was detected by targeting one gene with two or three guide RNAs, the established all-in-one platform enabled the simultaneous knockdown of ORF5 and ORF7 mRNAs by incorporating two corresponding guide protospacer sequences. Although the delivery efficiency of the all-in-one system still needs to be improved, the results showed the potential of CRISPR/Cas13-based multiplex RNA inference in mammalian cells.

PRRSV has a specific cell tropism^78^. *In vitro*, MARC-145 cells, derived from the African green monkey kidney cell line MA-104, display high susceptibility to PRRSV infection and are commonly used for PRRSV studies^79^. However, MARC-145 cells with a low chance of transfection (approximately 10% transfection efficiency by liposome lipid-based reagents, data not shown) did not sufficiently support CRISPR/Cas13 system delivery by an established all-in-one vector. To address this limitation, we employed an all-in-one lentiviral platform for the generation of stably expressing CRISPR/Cas13 MARC-145 cell lines. We observed high levels of PRRSV viral genomic knockdown in cells carrying a single crRNA. Notably, transgenic cells showed almost complete cleavage activities against genomic RNA and abrogated PRRSV infection with duplexed crRNAs targeting ORF5 and ORF7. The marked improvement in RNA splicing ability could be attributed to the lentiviral-mediated increase in robust and sustained expression of Cas13b and crRNAs compared to that observed after transient transfection. In addition, the establishment of CRISPR/Cas13 system expression prior to PRRSV infection favours the elimination of viral RNA. Nidovirus subgenomic RNAs are synthesized from the genomic RNA template by the discontinuous transcription mechanism^17,80^. The significant reduction in all PRRSV subgenomic RNAs in transgenic cells was mostly due to robust cleavage of viral genomic RNA. Since the splicing levels of PRRSV genomic RNA in the two single-crRNA cell lines were comparable, we further assessed the Cas13b cleavage specificity on PRRSV subgenomic RNAs. As the subgenomic RNAs shared the 3′ co-terminus, the 7-1 targeting region existed in all six subgenomic RNAs, whereas the 5-2 targeting region was absent in the two latter subgenomic RNAs. The qRT-PCR results showed significant differences that were detected in only subgenomic RNA 6 and 7 between the Cas13b-crRNA-5-2 and Cas13-crRNA-7-1 cell lines, demonstrating the specific viral subgenomic RNA knockdown ability of Cas13b. Since previous studies clearly indicated that no detectable off-target effects were caused by Cas13^38–40^, off-target effects were not addressed in the current study.

The current study represents the first application of CRISPR/Cas13 against mammalian RNA viruses in eukaryotic cells. However, there are still several challenges to implementing the CRISPR/Cas13 system to interfere with RNA virus infections *in vivo*. Although the lentivirus-mediated CRISPR/Cas delivery system prevented RNA virus infections in transgenic cells, the integration of exogenous genes into the host genome, especially in livestock animals, is a cause for concern in terms of food safety and public perception. The adeno-associated virus (AAV) system could be a potential alternative, but the large size of Cas effectors limits the packaging efficiency in AAV. The most recently identified type VI-D CRISPR effector Cas13d, the smallest class II Cas protein in mammalian cells, can be packaged into the AAV system and be potentially delivered into natural viral hosts^38^. CRISPR/Cas9-mediated dsDNA virus targeting may introduce mutations, insertions or deletions at guide RNA targeting sites leading to virus variants that might be more pathogenic or resistant to CRISPR/Cas9-directed disruption^81^. Although virus escape mutants can be restricted by simultaneously targeting multiple sites, this is an obstacle to CRISPR/Cas9 application in antiviral therapy^53^. For CRISPR/Cas13, the latest research demonstrated that no crRNA target site mutations were observed following Cas13 cleavage over a course of 48 h, suggesting a low likelihood of production of mutations by Cas13 targeting^73^. However, further work is needed to comprehensively assess the possibility of introducing virus variants by CRISPR/Cas13 cleavage in the long term. Once the formation of virus escape mutants becomes unavoidable, how many target sites (or genes) will be required to limit virus variants and prevent virus evolution. PRRSV comprises several structural proteins and NSPs. In our study, ORF 5 and ORF 7 have been proven to be ideal targets for CRISPR/Cas13 cleavage. However, other genes especially the genes coding NSPs that are essential for PRRSV transcription and replication will need to be investigated in the future.

Overall, the prospect of developing a CRISPR/Cas13 system to directly knockdown viral RNA represents an exciting avenue in antiviral therapy. Our study indicated that the CRISPR/Cas13b system can effectively knockdown the PRRSV genome *in vitro*, and can potentially be a potent therapeutic antiviral strategy. The impact of the current study will not be limited to PRRSV, as the results have a wider significance, and this study provides the foundation for studies about other RNA viruses infecting humans and animals.

## Methods

### Cell lines, viruses and plasmids

HEK293T, HEK293FT and MARC-145 cells were obtained from the American Tissue Collection Center (ATCC, USA) and cultured in Dulbecco’s Modified Eagle’s Medium (DMEM, Gibco) supplemented with 10% foetal bovine serum (FBS; Gibco) and 1% penicillin-streptomycin (Gibco). PRRSV-2 atypical strain 10PL01^82^ and typical strain 01NP1 were kindly provided by Chulalongkorn University Veterinary Diagnostic Laboratory (CU-VDL; Bangkok, Thailand) and propagated in MARC-145 cells using DMEM supplemented with 2% FBS. The viral titers were calculated by the Reed-Muench method^83^ and expressed as a 50% tissue culture infective dose (TCID_50_). PspCas13b fused with the HIV Rev gene NES expression vector (Addgene 103862) and PspCas13b guide RNA expression backbone with the U6 promoter (Addgene 103854) were generous gifts from Feng Zhang^40^. The eGFP reporter vector pEGFP-N1 was obtained from Clontech (Japan). The RFP657 reporter vector pTagRFP657-N1 was a gift from Vladislav Verkhusha (Addgene 31959)^84^. The lentiviral packaging plasmid pCMV-dR8.2 dvpr (Addgene 8455) and the VSV-G envelope plasmid pMD2.G (Addgene 12259) were gifts from Sheila A. Stewart^85^ and Didier Trono, respectively.

### Plasmid constructs

To generate the PRRSV GP5-eGFP, N-eGFP, GP5-RFP657 and N-RFP657 reporter constructs, PRRSV viral RNA was purified from PRRSV-infected MARC-145 cells, and cDNA was synthesized. Subsequently, the ORF5 and ORF7 genes were amplified by specific primers and inserted into the pEGFP-N1 and pTagRFP657-N1 vectors, respectively. To construct individual ORF5 and ORF7 reporters, the CMV promoter and ORF5 or ORF7 gene were inserted into the PUC19 vector. For the Cas13b expression plasmid, the EF-1α promoter and PspCas13b fused with the HIV Rev gene NES and an HA tag were cloned into the PUC19 vector. To establish a delivery vector carrying the Cas13b protein and streamline duplexed guide RNA cassettes, the eGFP reporter gene was amplified from the pEGFP-N1 vector and inserted downstream of Cas13b-NES-HA linked by a T2A skipping peptide. Two *BsmB* I restriction sites with different overhangs were introduced upstream of the EF-1α promoter to facilitate Golden Gate cloning of the guide RNA component cassettes. Next, Cas13b guide RNA backbones were modified by the introduction of *BsmB* I restriction sites with various overhangs. To generate the lentiviral transfer gene plasmid, the all-in-one vector was further modified by replacing eGFP with the puromycin gene and introducing the HIV genes 5′LTR, WPRE, cPPT/CTS and RRE. All primers are reported in Table S1.

### Guide cloning for the Cas13b system

The sense and antisense guide RNA oligonucleotides were synthesized (Table 1) with overhangs (F overhang is CACC and R overhang is CAAC). Each pair of oligonucleotides was annealed and phosphorylated with T4 PNK (NEB, USA) in a thermocycler using the following parameters: 37 °C for 30 min, 95 °C for 5 min and then ramped down to 25 °C at 5 °C/min. The modified guide expression backbones were digested with Bbs I (NEB, USA) for 30 min at 37 °C. Subsequently, the phosphorylated and annealed oligo duplex was ligated into the corresponding Bbs I-digested guide expression backbone by Quick Ligase (NEB, USA) at room temperature for 10 min. Then, NEB 5α competent E. coli cells (NEB, USA) were transformed using 2 μl of the assembled product. The plasmids were extracted from overnight culture using a Plasmid Miniprep Kit (MN, Germany) and verified by sequencing. Finally, a mixture of the constructed guide RNA backbone and Cas13b destination plasmid was treated with BsmB I (NEB, USA) and T4 DNA ligase (NEB, USA) in a thermocycler with the following program: 30 cycles of 5 min at 37 °C and 5 min at 16 °C followed by 5 min at 60 °C. After transformation into One Shot Stbl 3 chemically-competent *E. coli* cells (Transgene, China), the plasmids were prepared by a Plasmid Midiprep Kit (Qiagen, Germany).

### Transfection for PRRSV RNA knockdown

To assess PspCas13b activities in eukaryotic cells, approximately 1 × 10^4^ HEK293T cells per well were plated into 24-well plates 16 h prior to transfection to ensure 60–70% confluency at the time of transfection. Transient transfections were performed using Lipofectamine 3000 (Invitrogen). For triple-plasmid transfection, cells were transfected with 250 ng of Cas13b vector, 500 ng of guide RNA plasmid and 250 ng of eGFP reporter plasmid. For dual-plasmid transfection, cells were transfected with 500 ng of all-in-one Cas13b-gRNA plasmid along with 500 ng RFP657 reporter plasmid.

### Fluorescence assay

To determine the expression of reporter genes after CRISPR/Cas13b activity along with guide RNA targeting, transfected HEK293T cells were fixed in 3.7% formaldehyde for 10 min at room temperature and then permeabilized with 0.2% Triton X-100 in PBS for 10 min. Subsequently, Fluoroshield mounting medium with DAPI (Abcam, USA) was dropped onto the cells, and imaging was performed by a Olympus IX73 inverted microscope with cellSens Dimensions imaging software (Olympus, Japan).

### Flow cytometry analysis

At 48 h post-transfection, cells were dissociated by TrypLE^TM^ Select (Invitrogen, USA) and washed once with cold PBS by centrifugation for 5 min at 1,000 × g and 4 °C. The supernatant was removed by aspiration, and the cells were resuspended in 500 μl of FACS buffer (PBS supplemented with 0.5% BSA and 0.1% sodium azide). The cells were analysed using a Cytomics FC 500 MPL flow cytometry system (Beckman Coulter, USA). The data were analysed using MXP software (Beckman Coulter, USA). To select MARC-145 cells stably expressing Cas13b after infection with lentivirus, harvested cells were fixed in 4% paraformaldehyde and permeabilized with ice-cold 90% methanol. After washing, cells were stained with a 1:50 dilution of an HA tag antibody (Thermo Fisher). For secondary staining, a 1:100 dilution of goat anti-mouse IgG1-FITC was added to cells and incubated in the dark. For each measurement, at least 10,000 events were gated.

### Quantitative RT-PCR

Total RNA from cultured cells was purified by the GenUP^TM^ Total RNA Kit (Biotechrabbit, Germany) with DNase treatment following the manufacturer’s instructions. The quality of the RNA was determined by a NanoDrop 1000 (Thermo Fisher, USA). The first-strand cDNA was synthesized by M-MuLV reverse transcriptase (NEB, USA). Quantitative PCR was performed with Luna^®^ Universal qPCR Master Mix (NEB, USA) using a StepOnePlus^TM^ Real-Time PCR System (Applied Biosystems, USA). The amplification reaction consisted of an initial denaturation at 95 °C for 1 min, followed by 45 cycles of denaturation at 95 °C for 15 s and extension at 60 °C for 30 s. The primers used for amplification are shown in Table 1. Primer specificity was confirmed by melting curve analysis. The relative fold changes were quantified using the delta delta C_t_ method with glyceraldehyde-3-phosphate dehydrogenase (GAPDH) as the endogenous control for normalization.

### Generation of CRISPR/Cas13b-gRNA expressing cell lines

HEK293FT cells were plated into 10 mm plates and transfected with 5 µg of the constructed lentiviral transfer plasmid, 1.25 µg of pMD 2. G and 2.5 µg of pCMV-d8.2 dvpr by Lipofectamine 3000 according to the manufacturer’s instructions. At 6 h post-transfection, the packaging medium was replaced. Viral supernatants were collected 24 h and 52 h after medium replacement, pooled and clarified through a 45 μm PVDF filter (Millipore). The supernatants were then concentrated by Amicon Ultra-15 centrifugal filter units. For lentiviral transduction, MARC-145 cells were transduced with concentrated lentiviral supernatants supplemented with 8 μg/ml polybrene (Millipore). At 72 h post-infection, cells were screened via puromycin (6 μg/ml) treatment. After 3 weeks of selection, single clones were isolated by limiting the dilution. Successfully transduced cells expressing Cas13b and the corresponding guide RNA were detected by flow cytometry and PCR, respectively.

### Infection of CRISPR/Cas13b-MARC-145 cells with PRRSV

For the virus inhibition assay, MARC-145 and parental MARC-145 cells with stable expression of CRISPR/Cas13b-gRNA were cultured overnight in a 12-well plate and then infected with PRRSV 10PL01 or 01NP1 at various MOIs. After 1 h of incubation at 37°C, non-adherent viruses were aspirated, and cells were replenished with DMEM containing 2% FBS. Cell supernatants were collected at different time points, and virus titers were determined. Cells were scraped from the wells in PBS and subjected to qRT-PCR to determine viral genomic and subgenomic RNA levels.

### Statistical analysis

All experiments were performed three times. Statistical significance was calculated using two-way ANOVA to determine the differences between the CRISPR/Cas13-targeted group and the control group. All statistical analyses were performed using GraphPad Prism software (version 8.0, San Diego, CA, USA).

## Supplementary information

Supplementary materials.
