# Supplementary material for: Abrogation of PRRSV infectivity by CRISPR-Cas13b-mediated viral RNA cleavage in mammalian cells
Source: Sci Rep. 2020 Jun 15;10:9617. doi: 10.1038/s41598-020-66775-3 (PMC7295971; doi:10.1038/s41598-020-66775-3)
Supplement: Supplementary file 1 — Supplementary materials. [file 41598_2020_66775_MOESM1_ESM.pdf]

Supplementary information

Abrogation of PRRSV infectivity by CRISPR-Cas13b-mediated viral RNA cleavage in mammalian cells

Jin Cui, Navapon Techakriengkrai, Teerawut Nedumpun, Sanipa Suradhat*

Department of Veterinary Microbiology, Faculty of Veterinary Science, Chulalongkorn University, Pathumwan, Bangkok 10330, Thailand

* Correspondence: Sanipa Suradhat, Department of Veterinary Microbiology, Faculty of Veterinary Science, Chulalongkorn University, Pathumwan, Bangkok 10330, Thailand.

E-mail: Sanipa.S@chula.ac.th

Supplementary Table 1. Primer used in this study

**Primer Name**	**Sequence (5′ to 3′)**
EGFP-N1-ORF5-F	CCGGAATTCTGATGTTGGGGAAGTGCTTGACCGCG
EGFP-N1-ORF5-R	CGGGATCCCGGAGACGACCCCATTGTTCCGCTGA
EGFP-N1-ORF7-F	CCGGAATTCTGATGCCAAATAACAACGGCAAGCA
EGFP-N1-ORF7-R	CGGGATCCCGTGCTGAGGGTGATGCTGTGGCGCGG
RFP657-ORF7-F	TTCGAATTCATGCCAAATAACAACGG
RFP657-ORF7-R	GGTGGATCCCGTGCTGAGGGTGATGCTG
RFP657-ORF5-F	TTCGAATTCATGTTGGGGAAGTGCTTG
RFP657-ORF5-R	GGTGGATCCCGGAGACGACCCCATTGTT
P2A+EGFP-NEB_F	AGCCGGAGATGTCGAAGAGAATCCTGGACCGGCCA CCATGGTGAGCAAGGG
P2A+EGFP-NEB_R	TGTTTCAGCAGAGAGAAGTTTGTTGCTCTAGAGACC GGTGGATCCCGGGC
PC0046_Xba I_NEB kit-F	AGCAGAATTCGATATCAAGCTTATCGATAATC
PC0046_Xba I_NEB kit-R	CTAGAGGCATAGTCGGGGACATC
PC0046-R2-BsbmI-Neb_F	GCAGAGATCCAGTTTGGTTCGTCTCCTTGTAATTAA TGCAAAGATGGATAAAG
PC0046-R2-BsbmI-Neb_R	TGTCCCTGTAACGTCTCTGTACTAAACCCGTAAACC CGAAAATTTTGAATTTTTG
P2A+EGFP-F	TGCTCTAGAGCAACAAACTTCTCTCTGCT
P2A+EGFP-R	CCGGAATTCTTACTTGTACAGCTCGTCCA
PC0043-R1-NEB-F	TTCACCGTCATCACCGAAACGCGCGACAGGAAAGGGCC TCGTGATACG
PC0043-R1-NEB-R	AACCTCTGACACATGCAGCTCCCGTACAGGGTCACAGCT TGTCTGTAAG
PC0043-R2-5’BsmBI-NEB-F	AGTACGAGGGCCTATTTCCCATG
PC0043-R2-5’BsmBI-NEB-R	GAGACGTGGCGTAATCATGGTCATAG
PC0043-R3-3’BsmBI-NEB-F	AGACGGGTACCGAGCTCGAATTC
PC0043-R3-3’BsmBI-NEB-R	CAACAAAAAAAAGTTGTAATAGCCCC
PC0043-R4-3’BsmBI-NEB-F	AGACGGGTACCGAGCTCGAATTC
PC0043-R3-3’BsmBI-NEB-R	CATATCAAAAAAGTTGTAATAGCCCC
PC0043-R5-5’BsmBI-NEB-F	CCACGTCTCAGATAGAGGGCCTATTTCCC
PC0043-R5-5’BsmBI-NEB-R	CGTAATCATGGTCATAGC

46R4-NEB-F	CTTGTGTTAATTAATGCAAAGATGGATAAAG
46R4-NEB-R	GAGACGCAAACTGGATCTCTGCTG
qPCR-nsp9-F2	CCTGCAATTGTCCGCTGGTTTG [1]
qPCR-nsp9-R2	GACGACAGGCCACCTCTCTTAG [1]
qPCR-Leader-F	CACCTTGCTTCCGGAGTTG [2]
qPCR-sgmRNA2-R	CAGCCAACCGGCGATTGTGAA [2]
qPCR-sgmRNA3-R	GCAAAGCGGGCATACCGTGT [2]
qPCR-sgmRNA4-R	ACGAAGTCTGATGCTGCGGTG [2]
qPCR-sgmRNA5-R	CTGGCGTTGACGAGCACAGCA [2]
qPCR-sgmRNA6-R	CATCACTGGCGTGTAGGTAATGGA [2]
qPCR-sgmRNA7-R	GGCTTCTCCGGGTTTTTCTTCCTA [2]
Monkey-GAPDH-F	ACCCACTCTTCCACCTTCGACGCT
Monkey-GAPDH-R	TGTTGCTGTAGCCAAATTCG
Human-GAPDH-F	GTCTCCTCTGACTTCAACAGCG
Human-GAPDH-R	ACCACCCTGTTGCTGTAGCCAA
PCR-crRNA-5-1-F	GACGACCCCATTGTTCCGCTGAAACTCTGG
PCR-crRNA-5-2-F	GCCAATCTGTGCCATTCAGCTCACATAGCG
PCR-crRNA-5-3-F	GCTGTTGCTGGCGTTGGCGAGCACAGCAAG
PCR-crRNA-7-1-F	ACACAATTGCCGCTCACTAGGGGTAAAGTG
PCR-crRNA-DR-R	GTTGTAATAGCCCCTCAAAACTGG

Fig S1

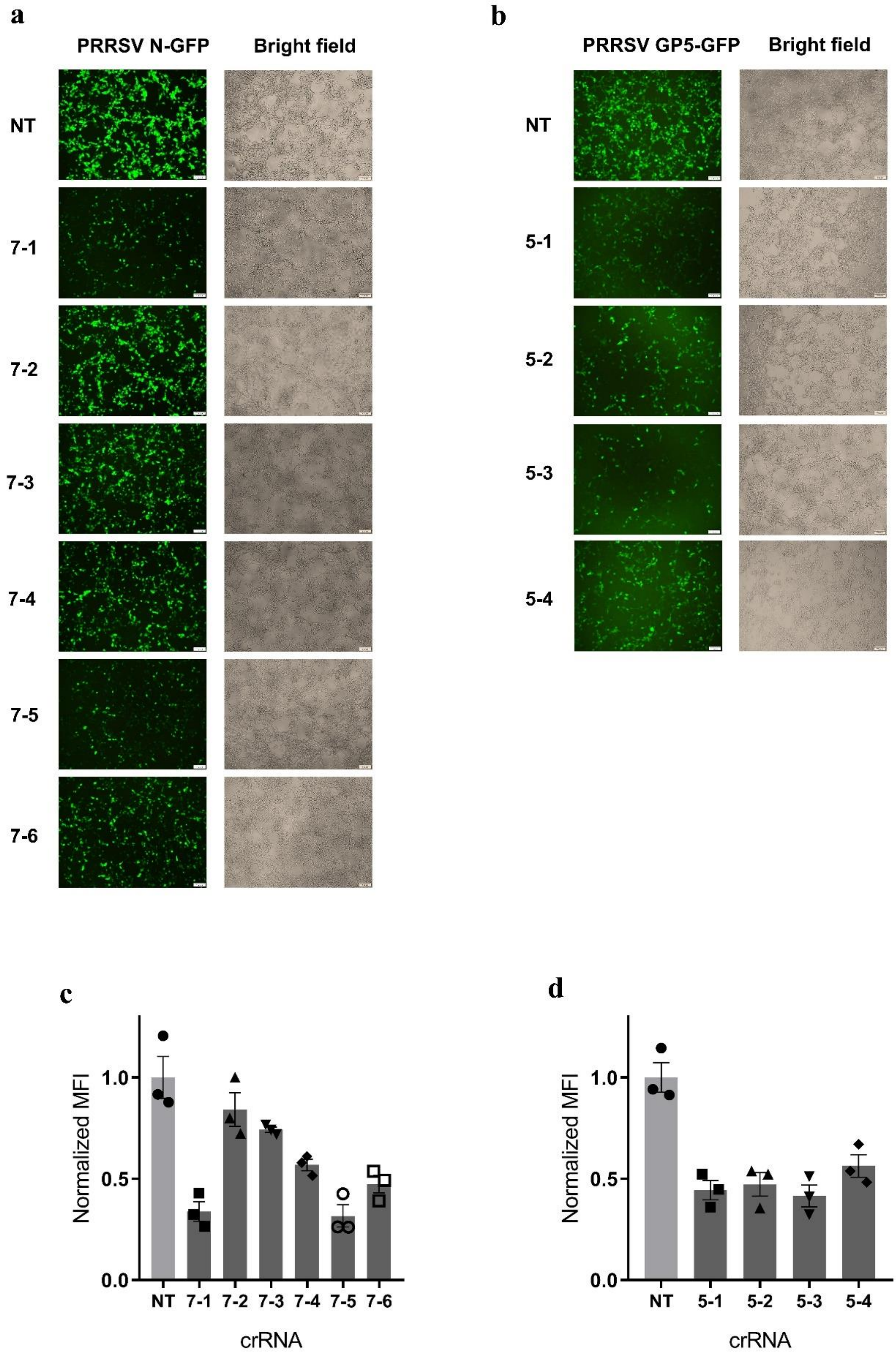
a
PRRSV N-GFP
Bright field
NT
7-1
7-2
7-3
7-4
7-5
7-6
b
PRRSV GP5-GFP
Bright field
NT
5-1
5-2
5-3
5-4
c
Normalized MFI
1.0
0.5
0.0
NT
7-1
7-2
7-3
7-4
7-5
7-6
crRNA
d
Normalized MFI
1.0
0.5
0.0
NT
5-1
5-2
5-3
5-4
crRNA

Fig S1 Determination of most potent crRNA targeting PRRSV ORF5 and ORF7 genes. Microscopic fluorescence images showing the expression of the PRRSV ORF7-eGFP (a) and ORF5-eGFP (b) reporters after CRISPR/Cas13b with various crRNA targeting. The bar indicates 100 μm. Relative expressions of PRRSV N (c) and GP5 (d) proteins were indicated as mean fluorescence intensity (MFI) normalized to NT crRNA control. Values shown as mean ± SEM with n = 3.

Fig S2

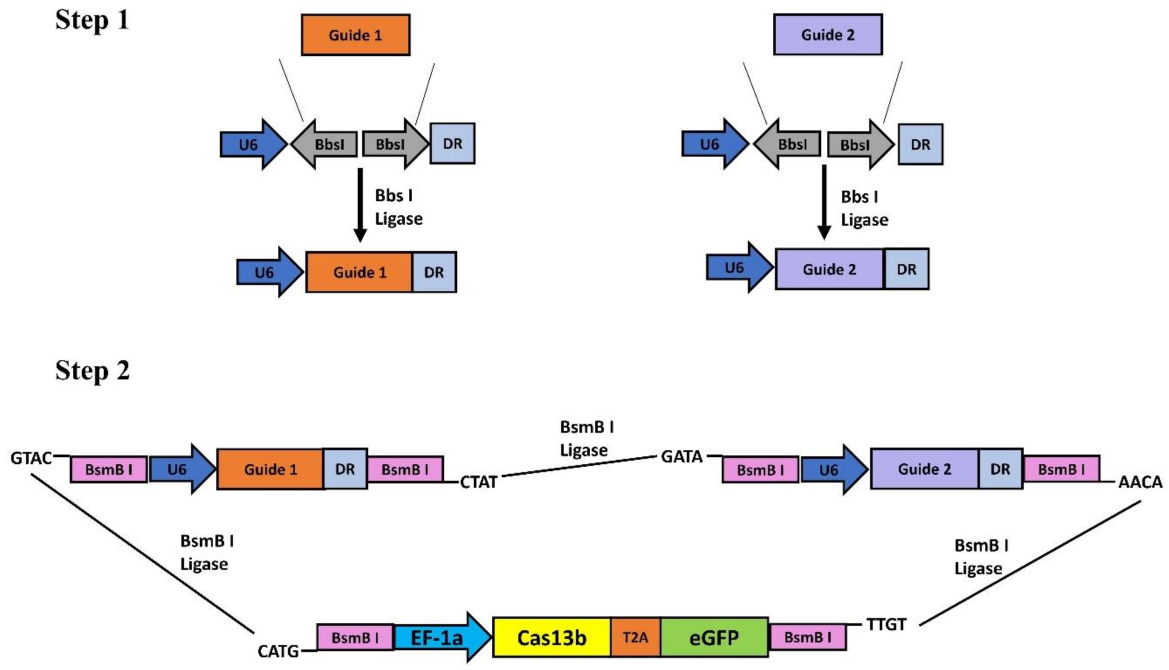

Fig S2 Steps for cloning guide RNA protospacer sequences into the all-in-one platform by Golden Gate Assembly. Step 1, oligonucleotides encoding guide RNA protospacer sequences were cloned into corresponding guide RNA backbones with the *Bbs I* restriction enzyme. Step 2, each guide RNA expression construct was subcloned into an established Cas13b effector vector with recognition by the type IIS restriction enzyme *BsmB I* with distinct overhangs for specific insertion.

Fig S3

a

b

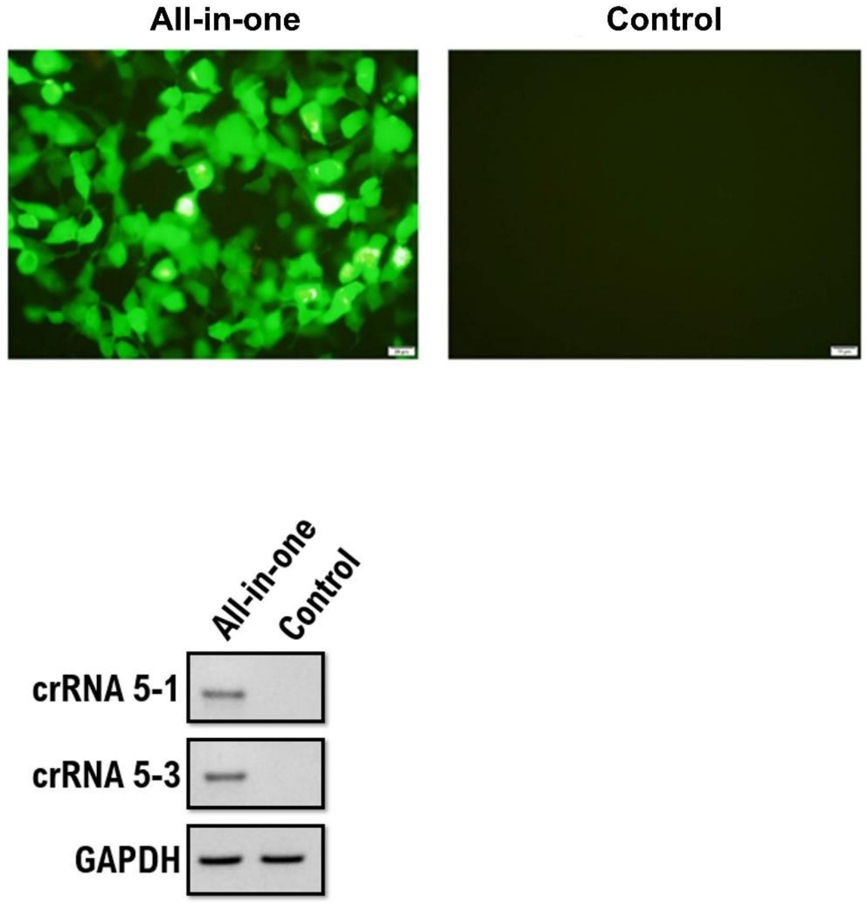

Fig S3 Validation of Cas13b and crRNAs expression by all-in-one delivery system. The crRNA 5-1 and 5-3 were inserted into the all-in-one cassettes and the constructed plasmid was transfected into HEK293T cells. (a) At 48 h post transfection, the Cas13b expression was assessed by IFA using antibody against HA tag fused to Cas13b protein. The pUC19 was set as a control. The bar indicates 50 μm. (b) The presence of 5-1 and 5-3 crRNAs were determined by PCR. The amplicons were separated in 5% agarose gel.

Fig S4

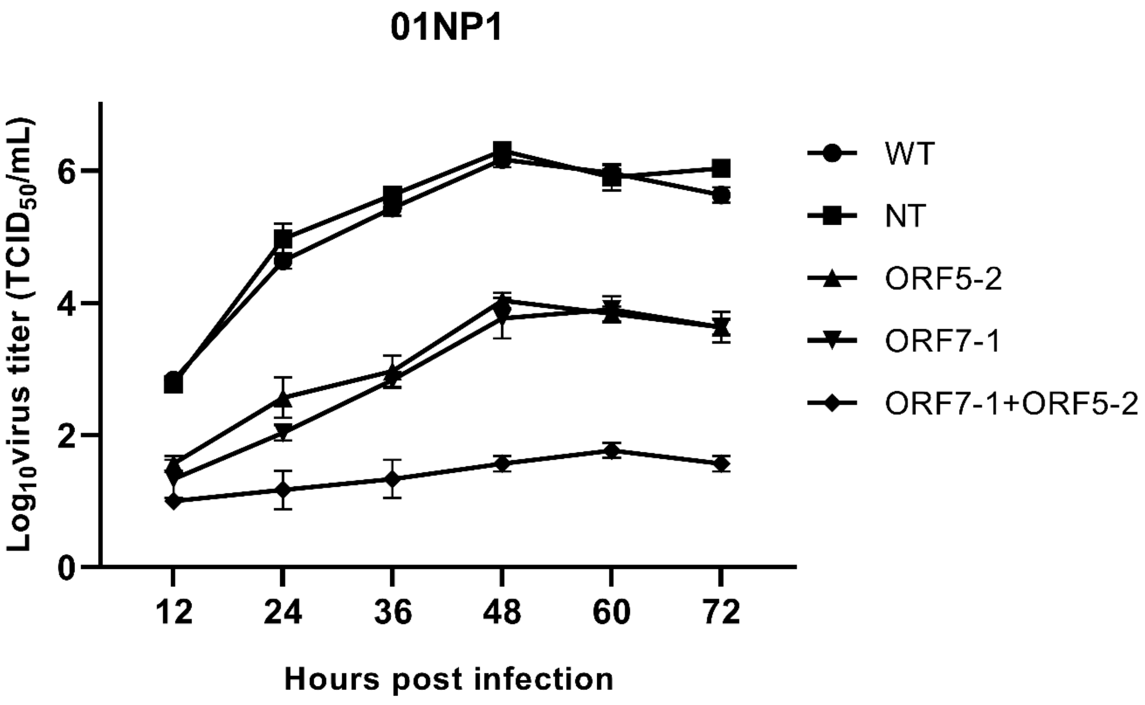

Fig S4 The growth kinetics of classic PRRSV strain 01NP1 in transgenic cells. The transgenic cells were infected with the classic PRRSV strain 01NP1 at an MOI of 0.1. Culture medium was harvested every 12 h, and the viral titers were calculated by the Reed-Muench method and expressed as a 50% tissue culture infective dose ($TCID_{50}$).

a

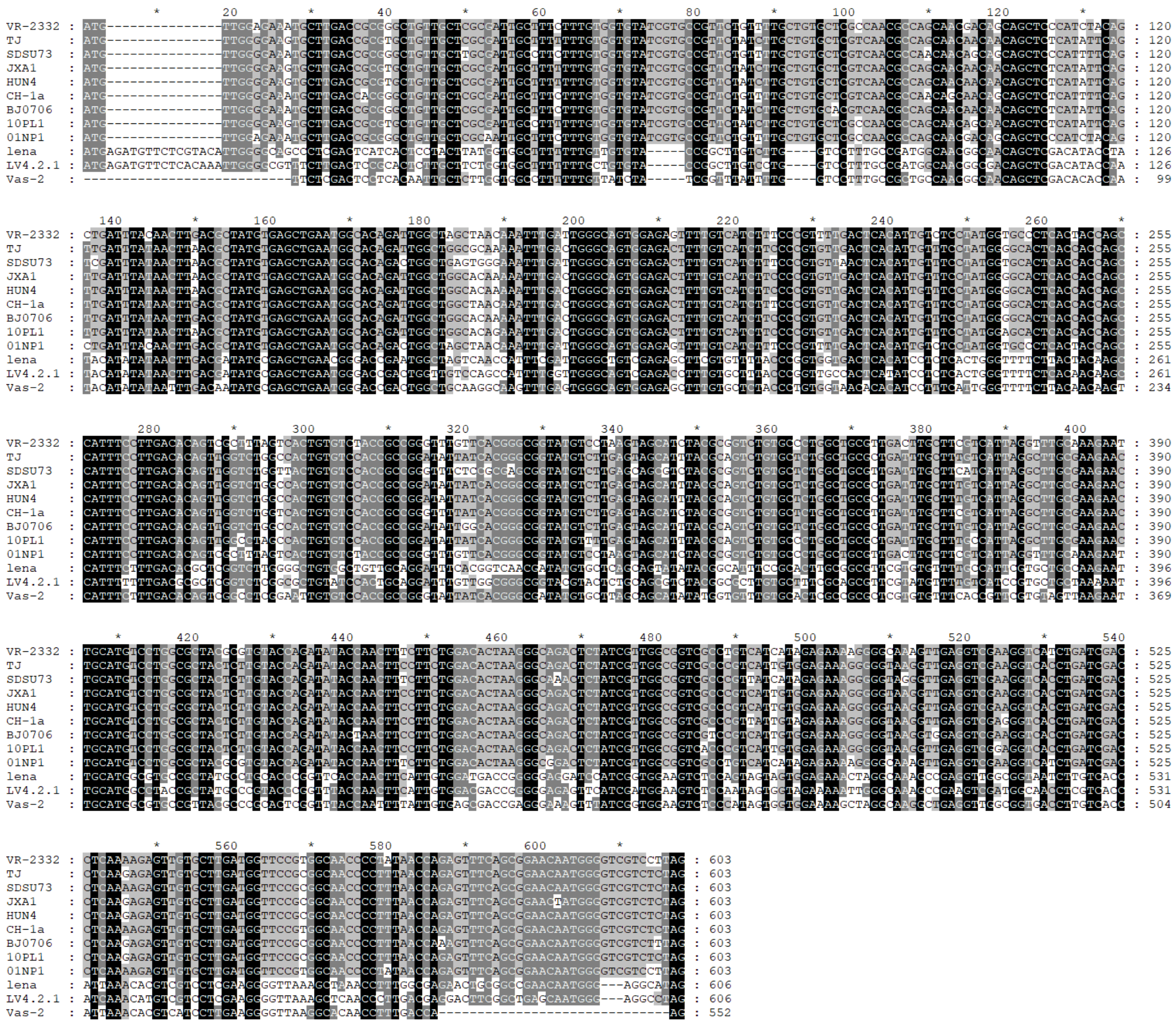
VR-2332
TJ
SDSU73
JXA1
HUN4
CH-1a
BJ0706
10PL1
01NP1
lena
LV4.2.1
Vas-2

b

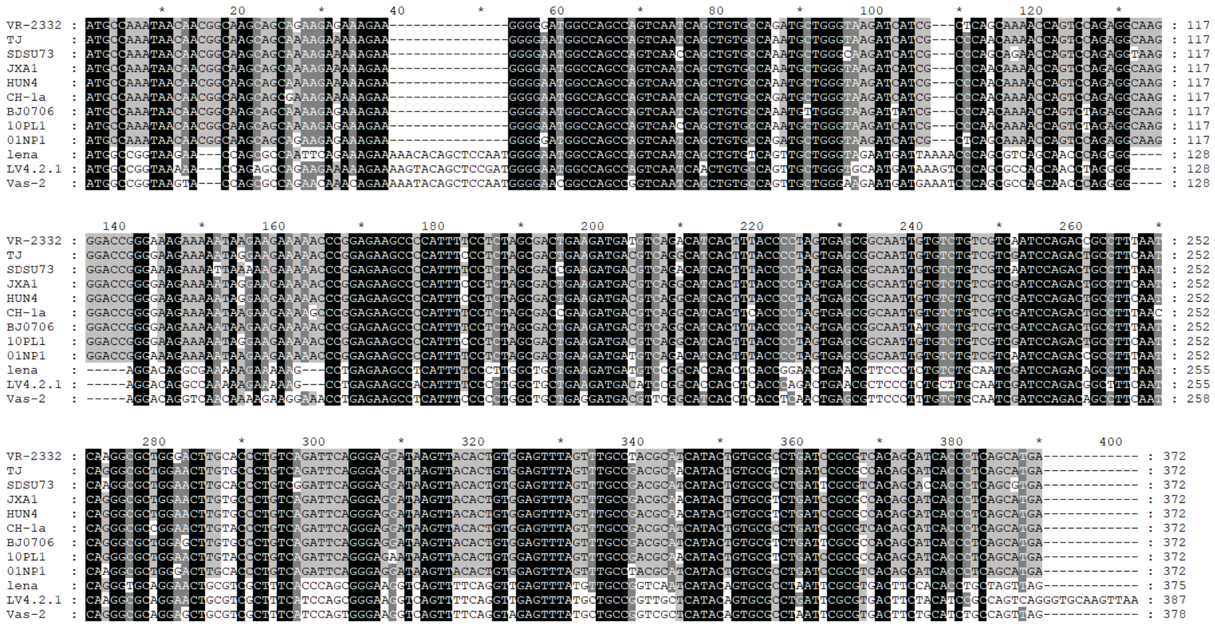
VR-2332
TJ
SDSU73
JXA1
HUN4
CH-1a
BJ0706
10PL1
01NP1
lena
LV4.2.1
Vas-2

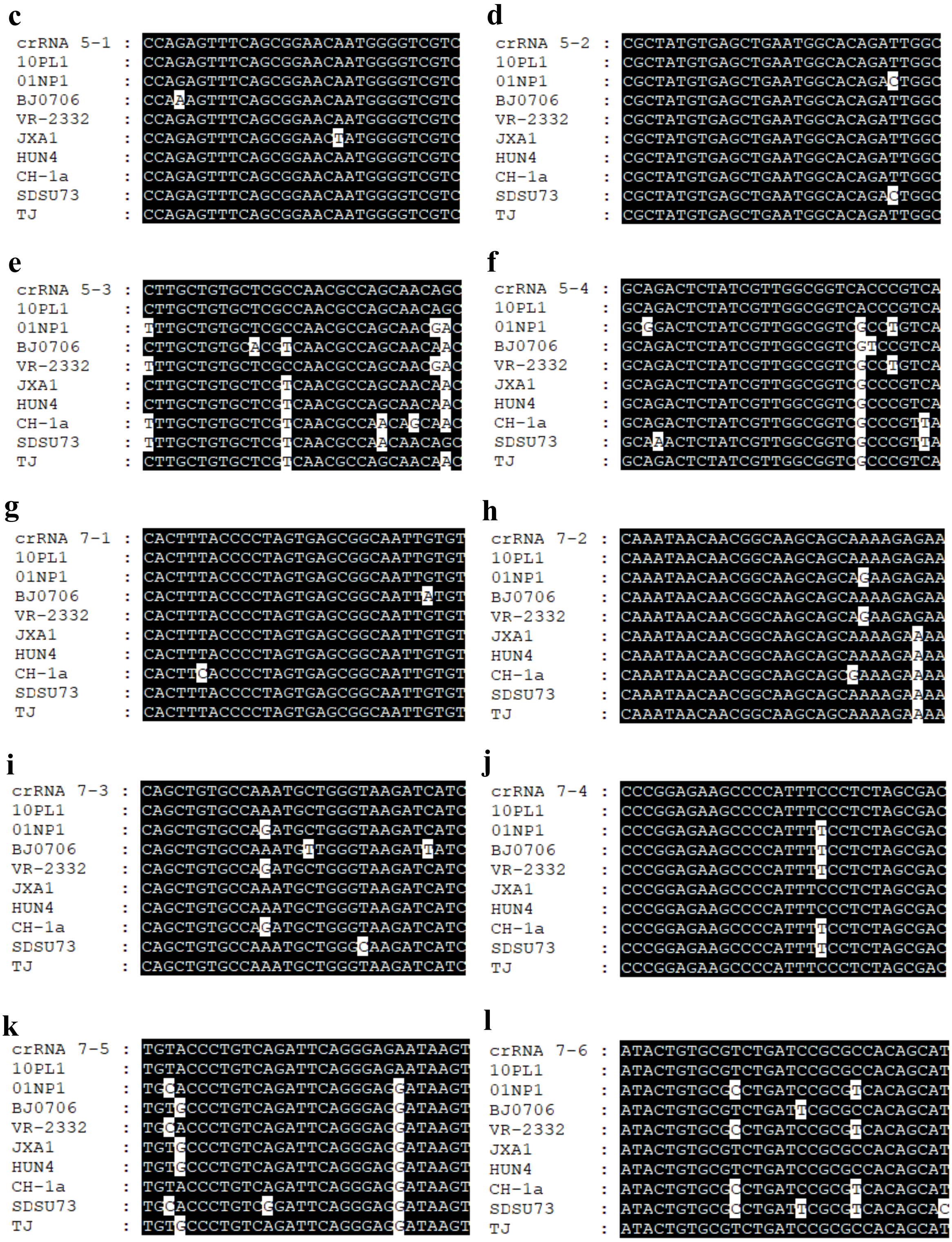
c
crRNA 5-1 : CCAGAGTTTCAGCGGAACAATGGGGTCGTC
10PL1 : CCAGAGTTTCAGCGGAACAATGGGGTCGTC
01NP1 : CCAGAGTTTCAGCGGAACAATGGGGTCGTC
BJ0706 : CCAAAGTTTCAGCGGAACAATGGGGTCGTC
VR-2332 : CCAGAGTTTCAGCGGAACAATGGGGTCGTC
JXA1 : CCAGAGTTTCAGCGGAACTATGGGGTCGTC
HUN4 : CCAGAGTTTCAGCGGAACAATGGGGTCGTC
CH-1a : CCAGAGTTTCAGCGGAACAATGGGGTCGTC
SDSU73 : CCAGAGTTTCAGCGGAACAATGGGGTCGTC
TJ : CCAGAGTTTCAGCGGAACAATGGGGTCGTC
d
crRNA 5-2 : CGCTATGTGAGCTGAATGGCACAGATTGGC
10PL1 : CGCTATGTGAGCTGAATGGCACAGATTGGC
01NP1 : CGCTATGTGAGCTGAATGGCACAGACTGGC
BJ0706 : CGCTATGTGAGCTGAATGGCACAGATTGGC
VR-2332 : CGCTATGTGAGCTGAATGGCACAGATTGGC
JXA1 : CGCTATGTGAGCTGAATGGCACAGATTGGC
HUN4 : CGCTATGTGAGCTGAATGGCACAGATTGGC
CH-1a : CGCTATGTGAGCTGAATGGCACAGATTGGC
SDSU73 : CGCTATGTGAGCTGAATGGCACAGACTGGC
TJ : CGCTATGTGAGCTGAATGGCACAGATTGGC
e
crRNA 5-3 : CTTGCTGTGCTCGCCAACGCCAGCAACAGC
10PL1 : CTTGCTGTGCTCGCCAACGCCAGCAACAGC
01NP1 : TTTGCTGTGCTCGCCAACGCCAGCAACGAC
BJ0706 : CTTGCTGTGCACGTCAACGCCAGCAACAAC
VR-2332 : TTTGCTGTGCTCGCCAACGCCAGCAACGAC
JXA1 : CTTGCTGTGCTCGTCAACGCCAGCAACAAC
HUN4 : CTTGCTGTGCTCGTCAACGCCAGCAACAAC
CH-1a : TTTGCTGTGCTCGTCAACGCCAACAGCAAC
SDSU73 : TTTGCTGTGCTCGTCAACGCCAACAACAGC
TJ : CTTGCTGTGCTCGTCAACGCCAGCAACAAC
f
crRNA 5-4 : GCAGACTCTATCGTTGGCGGTCACCCGTCA
10PL1 : GCAGACTCTATCGTTGGCGGTCACCCGTCA
01NP1 : GCGGACTCTATCGTTGGCGGTCGCCTGTCA
BJ0706 : GCAGACTCTATCGTTGGCGGTCGTCCGTCA
VR-2332 : GCAGACTCTATCGTTGGCGGTCGCCTGTCA
JXA1 : GCAGACTCTATCGTTGGCGGTCGCCCGTCA
HUN4 : GCAGACTCTATCGTTGGCGGTCGCCCGTCA
CH-1a : GCAGACTCTATCGTTGGCGGTCGCCCGTTA
SDSU73 : GCAAACTCTATCGTTGGCGGTCGCCCGTTA
TJ : GCAGACTCTATCGTTGGCGGTCGCCCGTCA
g
crRNA 7-1 : CACTTTACCCCTAGTGAGCGGCAATTGTGT
10PL1 : CACTTTACCCCTAGTGAGCGGCAATTGTGT
01NP1 : CACTTTACCCCTAGTGAGCGGCAATTGTGT
BJ0706 : CACTTTACCCCTAGTGAGCGGCAATTATGT
VR-2332 : CACTTTACCCCTAGTGAGCGGCAATTGTGT
JXA1 : CACTTTACCCCTAGTGAGCGGCAATTGTGT
HUN4 : CACTTTACCCCTAGTGAGCGGCAATTGTGT
CH-1a : CACTTCACCCCTAGTGAGCGGCAATTGTGT
SDSU73 : CACTTTACCCCTAGTGAGCGGCAATTGTGT
TJ : CACTTTACCCCTAGTGAGCGGCAATTGTGT
h
crRNA 7-2 : CAAATAACAACGGCAAGCAGCAAAAGAGAA
10PL1 : CAAATAACAACGGCAAGCAGCAAAAGAGAA
01NP1 : CAAATAACAACGGCAAGCAGCAGAAGAGAA
BJ0706 : CAAATAACAACGGCAAGCAGCAAAAGAGAA
VR-2332 : CAAATAACAACGGCAAGCAGCAGAAGAGAA
JXA1 : CAAATAACAACGGCAAGCAGCAAAAGAAAA
HUN4 : CAAATAACAACGGCAAGCAGCAAAAGAAAA
CH-1a : CAAATAACAACGGCAAGCAGCGAAAGAAAA
SDSU73 : CAAATAACAACGGCAAGCAGCAAAAGAAAA
TJ : CAAATAACAACGGCAAGCAGCAAAAGAAAA
i
crRNA 7-3 : CAGCTGTGCCAAATGCTGGGTAAGATCATC
10PL1 : CAGCTGTGCCAAATGCTGGGTAAGATCATC
01NP1 : CAGCTGTGCCAGATGCTGGGTAAGATCATC
BJ0706 : CAGCTGTGCCAAATGTTGGGTAAGATTATC
VR-2332 : CAGCTGTGCCAGATGCTGGGTAAGATCATC
JXA1 : CAGCTGTGCCAAATGCTGGGTAAGATCATC
HUN4 : CAGCTGTGCCAAATGCTGGGTAAGATCATC
CH-1a : CAGCTGTGCCAGATGCTGGGTAAGATCATC
SDSU73 : CAGCTGTGCCAAATGCTGGGCAAGATCATC
TJ : CAGCTGTGCCAAATGCTGGGTAAGATCATC
j
crRNA 7-4 : CCCGGAGAAGCCCCATTTCCCTCTAGCGAC
10PL1 : CCCGGAGAAGCCCCATTTCCCTCTAGCGAC
01NP1 : CCCGGAGAAGCCCCATTTTCCTCTAGCGAC
BJ0706 : CCCGGAGAAGCCCCATTTTCCTCTAGCGAC
VR-2332 : CCCGGAGAAGCCCCATTTTCCTCTAGCGAC
JXA1 : CCCGGAGAAGCCCCATTTCCCTCTAGCGAC
HUN4 : CCCGGAGAAGCCCCATTTCCCTCTAGCGAC
CH-1a : CCCGGAGAAGCCCCATTTTCCTCTAGCGAC
SDSU73 : CCCGGAGAAGCCCCATTTTCCTCTAGCGAC
TJ : CCCGGAGAAGCCCCATTTCCCTCTAGCGAC
k
crRNA 7-5 : TGTACCCTGTCAGATTCAGGGAGAATAAGT
10PL1 : TGTACCCTGTCAGATTCAGGGAGAATAAGT
01NP1 : TGCACCCTGTCAGATTCAGGGAGGATAAGT
BJ0706 : TGTGCCCTGTCAGATTCAGGGAGGATAAGT
VR-2332 : TGCACCCTGTCAGATTCAGGGAGGATAAGT
JXA1 : TGTGCCCTGTCAGATTCAGGGAGGATAAGT
HUN4 : TGTGCCCTGTCAGATTCAGGGAGGATAAGT
CH-1a : TGTACCCTGTCAGATTCAGGGAGGATAAGT
SDSU73 : TGCACCCTGTCGGATTCAGGGAGGATAAGT
TJ : TGTGCCCTGTCAGATTCAGGGAGGATAAGT
l
crRNA 7-6 : ATACTGTGCGTCTGATCCGCGCCACAGCAT
10PL1 : ATACTGTGCGTCTGATCCGCGCCACAGCAT
01NP1 : ATACTGTGCGCCTGATCCGCGTCACAGCAT
BJ0706 : ATACTGTGCGTCTGATTCGCGCCACAGCAT
VR-2332 : ATACTGTGCGCCTGATCCGCGTCACAGCAT
JXA1 : ATACTGTGCGTCTGATCCGCGCCACAGCAT
HUN4 : ATACTGTGCGTCTGATCCGCGCCACAGCAT
CH-1a : ATACTGTGCGCCTGATCCGCGTCACAGCAT
SDSU73 : ATACTGTGCGCCTGATTCGCGTCACAGCAC
TJ : ATACTGTGCGTCTGATCCGCGCCACAGCAT

Fig S5 The sequence alignments. The nucleotide alignments of ORF5 (a) and ORF7 (b) genes between PRRSV-1 and PRRSV-2 species. The conserved regions were highlighted in black colour. The nucleotide alignments of each crRNA and PRRSV-2 strains (c to l). The crRNAs were showed as reverse complement sequences. The conserved regions were highlighted in black colour and the mismatches between each crRNA and reference sequences were highlighted in white colour. The reference PRRSV strains used for alignments including PRRSV-1, subtype 1: LV4.2.1, subtype 2: Vas-2, subtype 3: Lena; PRRSV-2, typical strains: CH-1a, VR-2332 and 01NP1, atypical strains: BJ0706, JXA1, TJ, SDSU73, HUN4 and 10PL1. The 10PL1 and 01NP1 strains were the reference PRRSV-2 strains used in this study.

The full-length gel data

Supplementary information

Figure 4c

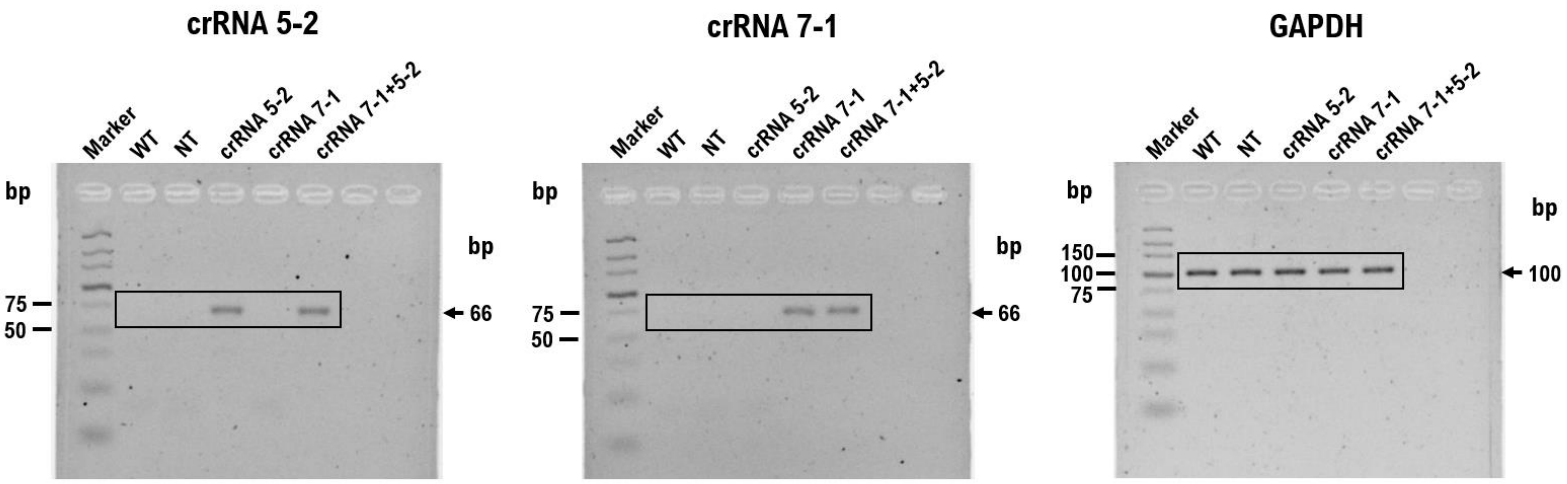

Figure S3b

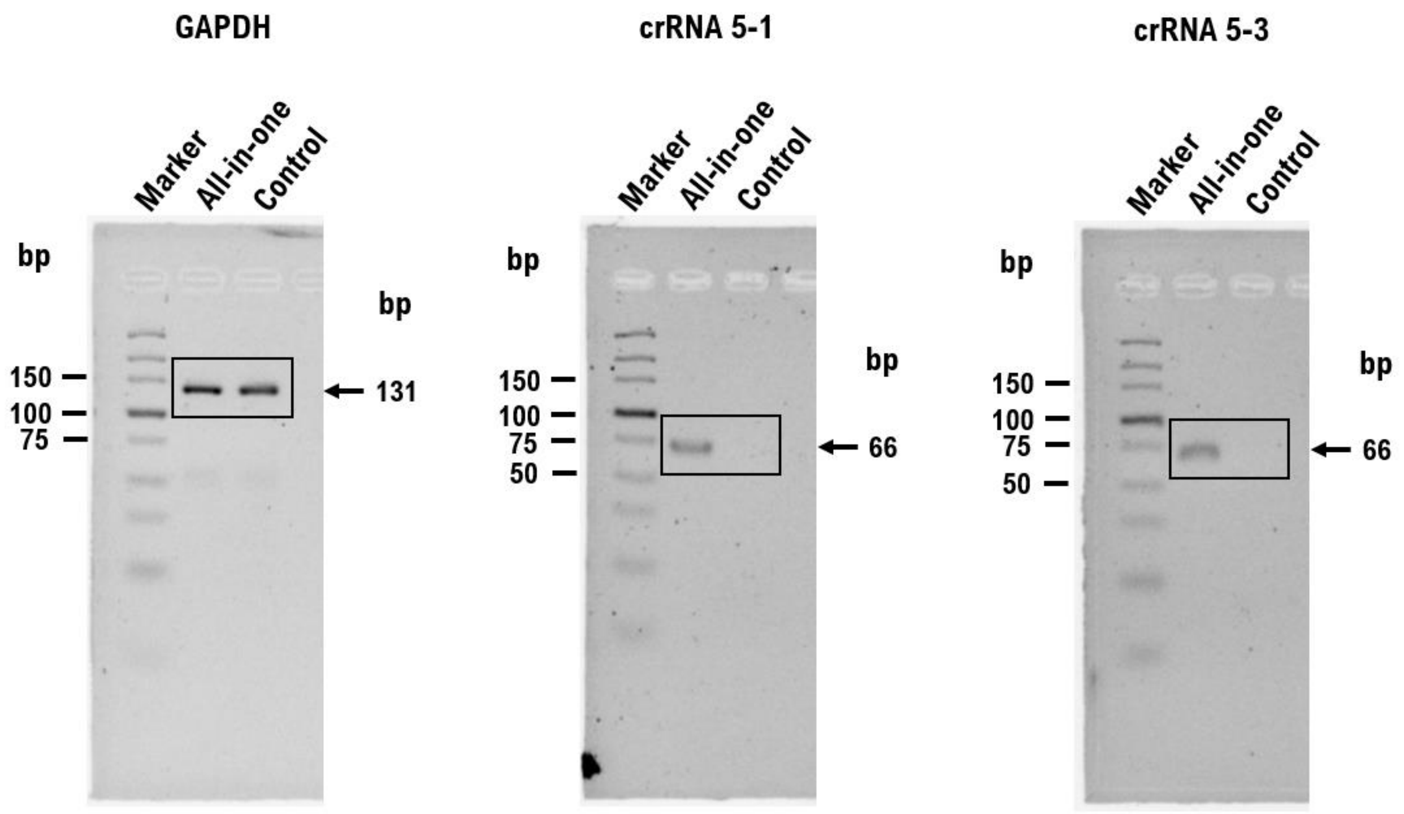

GAPDH
Marker
All-in-one
Control
bp
bp
150
100
75
131
crRNA 5-1
Marker
All-in-one
Control
bp
bp
150
100
75
50
66
crRNA 5-3
Marker
All-in-one
Control
bp
bp
150
100
75
50
66

References

1 Spear, A. & Faaberg, K. S. Development of a genome copy specific RT-qPCR assay for divergent strains of type 2 porcine reproductive and respiratory syndrome virus. *J Virol Methods* **218**, 1-6, doi:10.1016/j.jviromet.2015.02.007 (2015).

2 Liu, L. *et al.* Porcine Reproductive and Respiratory Syndrome Virus Nucleocapsid Protein Interacts with Nsp9 and Cellular DHX9 To Regulate Viral RNA Synthesis. *J Virol* **90**, 5384-5398, doi:10.1128/JVI.03216-15 (2016).
